# Chronotherapeutic administration of neoadjuvant chemotherapy reduces cancer-related fatigue-like behavior

**DOI:** 10.1016/j.bbih.2026.101262

**Published:** 2026-05-19

**Authors:** Claire O. Kisamore, Caleb A. Kisamore, Jayla M. Boyd, Paul J. Owolabi, Divya B. Kadri, Alan D. Mizener, Emidio E. Pistilli, William H. Walker

**Affiliations:** a West Virginia University Department of Neuroscience, Rockefeller Neuroscience Institute, Morgantown, WV, USA; b West Virginia University Cancer Institute, Morgantown, WV, USA; c West Virginia University Department of Exercise Physiology, Morgantown, WV, USA

**Keywords:** Circadian rhythms, Cancer-related fatigue, Voluntary wheel-running activity, Adverse events, Chemotherapy, Breast cancer

## Abstract

Circadian rhythms are ∼24-h oscillations in physiology and behavior and can be used to optimally time treatment, a practice known as chronotherapy. Chronotherapy for cancer treatment was first successfully utilized >50 years ago and has been demonstrated to improve anti-tumor efficacy and reduce toxic effects of chemotherapy. However, to our knowledge, no study has assessed chrono-chemotherapy for cancer-related fatigue (CRF). The most commonly reported side effect of chemotherapy is CRF, which peaks during treatment but can last up to a decade or longer in some patients. Although not specific to one cancer type, CRF is especially prevalent in patients undergoing treatment for breast cancer. We hypothesized that chronotherapy could reduce CRF in a mouse model of primary breast cancer. Female C57Bl/6 mice were orthotopically injected with the syngeneic EO771 mammary tumor cell line and housed individually with running wheels to assess fatigue-like behavior. Two doses of chemotherapy (paclitaxel or doxorubicin/cyclophosphamide [AC]) spaced 14 days apart were administered intraperitoneally at either the mid-inactive (*Zeitgeber* time [ZT] 6) or mid-active phase (ZT18). Mice receiving paclitaxel or AC at ZT6 demonstrated reduced fatigue-like behavior following the first dose compared to those receiving chemotherapy at ZT18. Central inflammatory markers were assessed following the second doses. Inflammatory profiles (composite z-scores) were higher in mice treated at ZT18 with paclitaxel but not AC. These data provide evidence that chronomodulation of neoadjuvant chemotherapy can reduce fatigue-like behavior in a mouse model of primary breast cancer and may inform clinical research to reduce adverse events in patients.

## Highlights

• Paclitaxel at ZT6 reduced fatigue-like behavior compared to treatment at ZT18.
• Response to AC affects chronotherapy benefit on tumor burden and FLB.
• AC at ZT6 reduced FLB in relative high responders compared to treatment at ZT18.

## 1 Introduction

Circadian rhythms, ∼24-h oscillations in physiology and behavior, play a role in virtually all aspects of bodily processes, including immunity, metabolism, and the cell cycle (Ince et al., 2023; Ozturk et al., 2017; Sulli et al., 2019). These rhythms are set by the suprachiasmatic nucleus (SCN), a bilateral structure in the hypothalamus known as the central clock. The SCN receives signals known as *zeitgebers* (German, meaning time-giver) that synchronize internal biological processes to the external 24-h day. In mammals, the most potent *zeitgeber* is light (optimally blue-wavelength range, ∼480 nm), which excites the photopigment melanopsin within intrinsically photosensitive retinal ganglion cells (ipRGCs). This initiates a signaling cascade via the retinohypothalamic tract activating the SCN, which in turn sets peripheral clocks accordingly (Laothamatas et al., 2023).

Circadian rhythms can be utilized to time therapeutic administration to improve efficacy (Rivard et al., 1993; Hrushesky and Bjarnason, 1993; Lévi et al., 1994; Abusamak et al., 2025; Shang and Li, 2023) or reduce side effects (Ting et al., 2015; Lévi et al., 1990; Gou et al., 2018; Hrushesky, 1985; Lévi et al., 1997; Li et al., 2015; von Roemeling and Hrushesky, 1989; Abusamak et al., 2025; Shang and Li, 2023), a practice known as chronotherapy. Chrono-chemotherapy (optimally timed administration of chemotherapy) was first demonstrated to be effective in the clinic over fifty years ago (Halberg et al., 2006) and has since been demonstrated to improve anti-tumor efficacy in acute lymphoblastic leukemia (Rivard et al., 1993), large B cell lymphoma (Kim et al), non-small cell lung cancer (Shang and Li, 2023), hepatoma (Akyel et al., 2025b), metastatic colorectal cancer, and other cancer types (Lévi et al., 1994). Similar beneficial effects of chronotherapy have been reported preclinically. Indeed, preclinical studies have demonstrated improved anti-tumor efficacy in models of leukemia (Haus et al., 1972), osteosarcoma (Granda et al., 2002), and breast cancer (Koritala et al., 2022). In addition, chronotherapy can also reduce adverse events in patients such as bleeding (Hrushesky, 1985), infection (Hrushesky, 1985; Kim et al), neutropenia (Chen et al., 2013; Kim et al), leukopenia (Chen et al., 2013; Shang and Li, 2023), nausea/vomiting (Chen et al., 2013; Shang and Li, 2023), diarrhea (Price et al., 2004), and neuropathy (Lévi et al., 1997) associated with chemotherapy. For example, a study conducted in patients with metastatic colorectal cancer administered a regimen of fluorouracil, oxaliplatin, and folinic acid at either a constant rate infusion or a chronomodulated infusion (i.e., each drug was given at a higher dose at a time previously demonstrated to be most highly tolerated) over the course of five days. After 12 courses (16 days between each course), only 16% of patients receiving the chronomodulated regimen reported peripheral neuropathy while 31% of patients receiving the constant rate infusion reported this toxic adverse effect (Lévi et al., 1997). Another notable instance is a phase I clinical trial of oxaliplatin, which was abandoned due to “practically constant” gastrointestinal toxicity (World Health Organization [WHO] grade 3-4 in 53% of participants) and dose-limiting sensory neuropathy following 75% of courses, which caused disabling neurotoxicity (WHO grade 3) in six out of 44 (13.6%) patients (Extra et al., 1990). Oxaliplatin was later tested successfully using a chronomodulated schedule (5-day infusion with peak dose at 16:00) (Caussanel et al., 1990), after which this regimen was validated in phase II and III clinical trials (Lévi et al., 1997; Lévi et al., 1992; Giacchetti et al., 2006). More recent pre-clinical data also demonstrate the crucial role of the circadian clock in the tolerance of oxaliplatin (Akyel et al., 2025a).

The most common adverse event associated with chemotherapy treatment is cancer-related fatigue (CRF; [Fabi et al., 2020; Kang et al., 2023]). CRF is defined by the National Comprehensive Cancer Network (NCCN) as “a distressing, persistent, subjective sense of physical, emotional, and/or cognitive tiredness or exhaustion related to cancer or cancer treatment that is not proportional to recent activity and interferes with usual functioning.” Common remedies for CRF include yoga (Lin et al., 2019), exercise (Cramp and Byron-Daniel, 2012), meditation (Haller et al., 2017), and limited pharmacological interventions (Mustian et al., 2017; Thong et al., 2020), but there is currently no approved pharmacologic treatment with consistent beneficial results (Mustian et al., 2017; Thong et al., 2020). This is in part due to the mechanism of CRF having yet to be fully elucidated. There are several hypotheses—one, deemed the “cytokine hypothesis of CRF,” proposes that peripheral/central inflammation (mostly driven by IL-6, IL-1β, and TNF-α [Bower, 2014; Schmidt et al., 2024; Song et al., 2024]) effect neural communication and the HPA axis (Bower, 2007; Bower and Lamkin, 2012; Hu et al., 2025), while other bodies of work reveal changes in skeletal muscle fatigue and metabolism thought to play a role in CRF (Bohlen et al., 2018; Dantzer et al., 2025). Although CRF is most commonly reported in patients undergoing treatment (Kang et al., 2023), up to 30% of survivors experience “chronic” CRF lasting years following completion of treatment (Fabi et al., 2020; Frick et al., 2017; Goldstein et al., 2006; Kröz et al., 2023). CRF can be caused by a tumor, treatment, or both (Thong et al., 2020), and is not specific to one type of treatment. However, CRF is reported in 80-90% of patients undergoing chemotherapy across cancer types (Fabi et al., 2020; Hofman et al., 2007). Rates of CRF, though, are seemingly high in breast cancer patients, especially during treatment (Fabi et al., 2020; Kang et al., 2023). Due to advancements in treatments, the survival rate for primary breast cancer is >90% in the United States (Tang et al., 2024). However, many survivors are left with debilitating quality-of-life problems, which commonly includes CRF, which can last up to a decade or longer in a substantial portion of patients (Bower et al., 2000; Bower et al., 2006; Thong et al., 2025). Although CRF is present across all treatment types (Kang et al., 2023), there is some evidence to suggest that chemotherapeutic regimens including anthracyclines (a class of chemotherapeutics that intercalate into the double helix of DNA and bind topoisomerase II to disrupt replication [Bayles et al., 2023]), specifically doxorubicin (Berger, 1998), lead to worse CRF-related outcomes (Berger, 1998; Kastelein et al., 2026). For example, a study of 1155 breast cancer patients concluded that subjects receiving anthracycline-based treatments reported significantly higher levels of fatigue, reduced activity in the first six months following treatment, and slower recovery time compared to subjects receiving non-anthracycline-based regimens (Kastelein et al., 2026). To our knowledge, there are no studies interrogating the use of chrono-chemotherapy to alleviate CRF. We therefore sought to determine if time of administration of AC (Adriamycin [doxorubicin]/cyclophosphamide) therapy or paclitaxel effects levels of CRF in a mouse model of primary breast cancer. Of note, AC therapy (a combination of an anthracycline and an alkylating agent) and paclitaxel (a taxane that forces apoptosis at the G2/M checkpoint by binding the β-tubulin subunit of microtubules [Abouzeid et al., 2025]), are recommended chemotherapeutics by the NCCN for the treatment of primary breast cancer. Given circadian regulation of drug toxicity and inflammation (Lévi et al., 2024) we hypothesized that optimally timed chemotherapy administration reduces CRF.

## 2 Methods

### 2.1 Animals

All procedures and experiments were approved by the West Virginia University Institutional Animal Care and Use Committee (protocol #2405077771). Female C57Bl/6 mice 6-8 weeks old were purchased from Charles River Laboratories (strain code 027) and allowed one week to acclimate prior to any experimental manipulation. Mice were provided *ad libitum* access to food (Teklad #2918) and water and maintained on a 12:12 light/dark cycle (ZT0 = lights on, ZT12 = lights off). *Zeitgeber* time (ZT) is circadian nomenclature used to denote time based on an entrainment cue (i.e., *zeitgeber*) and typically refers to the light cycle. In the present study, ZT0 is the time of lights on and ZT12 is the time of lights off (i.e., onset of activity). Following a week acclimation period mice were inoculated orthotopically with EO771 cells (detailed description below). Mice were administered either paclitaxel or a cocktail of doxorubicin/cyclophosphamide (AC) on days 8 and 22 following tumor inoculation. Tissue was collected on day 25 (paclitaxel, ZT6 n = 12, ZT18 n = 11) or 29 (AC, ZT6 n = 8, ZT18 n = 11). Tissue from mice that reached early removal criteria (ERC) of 20 mm tumor growth in any direction (measured externally with digital calipers), cachexia, or respiratory distress was not included. A total of 7 mice injected with paclitaxel and 11 injected with AC reached ERC prior to the end of the study. Of note, two cohorts of mice were run for each chemotherapeutic (i.e., paclitaxel and AC).

### 2.2 Tumors

The syngeneic C57Bl/6-derived EO771 triple negative mammary tumor cell line (Johnstone et al., 2015) was purchased from American Type Culture Collection (ATCC). Cells were cultured in Dulbecco's Modified Eagle Medium (DMEM; Gibco #10569) supplemented with 10% fetal bovine serum and 1x Antibiotic-Antimycotic (Gibco #15240). Cells were washed with Hank's Balanced Salt Solution (HBSS) and passaged using trypsin and resuspended in unsupplemented DMEM prior to injection. Mice were injected with 1x10^5^ cells (100 μl) orthotopically in the 9th mammary fat pad. Incisions were closed with tissue glue (Vetbond™) and mice were monitored daily until wounds were completely healed. All animals were administered 2 mg/kg OstiLox™ (meloxicam) at the time of surgery and 12 h post. Tumors were palpated five days following injection and measured with digital calipers every three days following palpation until reaching 15 mm in any direction, after which they were measured daily. Tumor volume was calculated as previously described using the equation tumor volume = (length × width^2^)/2 (Walker II et al., 2022).

### 2.3 Chemotherapy administration

Dose-dense AC therapy is the administration of 60 mg/m^2^ doxorubicin (Adriamycin) plus 600 mg/m^2^ cyclophosphamide every two weeks for 4 cycles followed by 80 mg/m^2^ paclitaxel every two weeks for 4 cycles and is a common standard of care for primary breast cancer recommended by the NCCN. Mice were intraperitoneally administered 55% of the human equivalent dose, based on body surface area (Reagan-Shaw et al., 2008), of either 14.4 mg/kg paclitaxel (Hospira) or a cocktail of 11 mg/kg doxorubicin (Pfizer) and 110 mg/kg cyclophosphamide (Amneal or Xgen).

### 2.4 Voluntary wheel running activity (VWRA)

Mice were singly housed with Low Profile Activity Wheels (Lafayette Instrument®) connected to the Wireless Gateway Receiver (Lafayette Instrument®). Activity was recorded using ClockLab Wireless Data Collection Program (Lafayette Instrument®). Mice were allowed an acclimation period of 17 days to the running wheels (Phan et al., 2024). Animals that failed to demonstrate normal activity patterns by the end of the acclimation period were removed from the study. VWRA was analyzed using ClockLab Analysis 6 (Lafayette Instrument®). Day one of the study started at ZT0 on the day following injection of tumor cells, therefore “days post inoculation” represent full cycles following inoculation. Bouts of activity were defined as periods of activity that did not go below 10 wheel revolutions/minute for more than 20 min at a time (Groot et al., 2019). VWRA of mice that reached ERC was used for analysis up until the day before collection/death. Therefore, the maximum sample size (depending on the day post inoculation) was 30 mice (ZT6 n = 15, ZT18 n = 15) for paclitaxel and 30 mice (ZT6 n = 15, ZT18 n = 15) for AC. However, due to mice reaching ERC, sample sizes at the end of the study were 23 for paclitaxel (ZT6 n = 12, ZT18 n = 11) and 19 for AC (ZT6 n = 8, ZT18 n = 11). Of note, a technical error in recording of wheel-running activity occurred after day 21 of one cohort receiving paclitaxel, therefore those data were unable to be reported.

### 2.5 Behavioral testing

Behavioral testing was completed during the active phase (i.e., after ZT12). Mice were allowed to acclimate to the room for 30 min prior to behavioral testing. A rest period of at least 10 min was allowed (in home cage) between each of the following behavioral tasks (listed in order of testing). Behavioral results of mice that reached ERC were included in analyses up until the day before collection/death.

#### 2.5.1 Grip strength

Maximum force of forelimb grip strength was measured using a Grip Strength Meter (Ugo Basile®). Mice were picked up by the tail and allowed to hold on to the grid on the grip strength apparatus with their forelimbs. They were then gently pulled backward until they let go of the grip, allowing for maximum grip strength output by the apparatus. Each animal underwent five trials and the average was used for data analysis. Normalized grip strength was calculated by dividing the average grip strength by the mass (g) of the mouse to normalize the effect of body mass on grip strength (Norden et al., 2014).

#### 2.5.2 Wire hang

Mice were placed on a 1 in. × 1 in. mesh grid (1.5 mm diameter) that was then inverted 35 cm above a padded surface. Latency to fall was recorded with a maximum limit of 300 s. Each animal underwent two trials with a 1-min rest period (in home cage) between trials.

#### 2.5.3 Rotarod

Mice were placed on a RotaRod (Ugo Basile®) and latency to fall (maximum limit 300 s) was recorded. Speed started at 4 RPM and accelerated to 40 RPM over 300 s. The test was repeated three times for each animal with a 15 min rest period (in home cage) between each trial.

### 2.6 Tissue collection

Prior to anesthesia, blood was collected via a submandibular bleed. Mice were placed under 3% isoflurane until unresponsive to toe pinch and then perfused transcardially with 1x PBS. Brains were quickly extracted and stored in RNA*later*™ Stabilizing Solution (Invitrogen). Tumors were extracted, weighed, and measured before storage in 10% formalin.

### 2.7 Quantitative reverse transcription polymerase chain reaction (qRT-PCR)

#### 2.7.1 qRT-PCR assay

Brains were stored in RNA*later*™ Stabilizing Solution (Invitrogen) and cortices and hypothalami were dissected. Tissue was dissociated mechanically and with QIAshredder (Qiagen LLC) columns. RNA was isolated using the RNeasy Plus Mini Kit (Qiagen LLC) per the manufacturer's instructions. cDNA was synthesized from RNA using SuperScript IV VILO Master Mix (Invitrogen) per the manufacturer's instructions. mRNA expressions of IL-6, TNF-α, and IL-1β were determined using Taqman probes (Mm00446190_m1, Mm00443258_m1, and Mm00434228_m1, respectively). Taqman Fast Advanced Master Mix (Invitrogen) was used in duplicate 20 μL reactions containing 40 ng of cDNA and Taqman probes for gene of interest and an 18s rRNA control. Cycling conditions were 95 °C for 20 s, 40x 3s cycles at 95 °C, and 60 °C for 30 s.

#### 2.7.2 qRT-PCR analysis

Relative gene expression was calculated using a relative standard curve and standardized to 18s rRNA expression (Borniger et al., 2017; Kang et al., 2018). Samples from mice that reached ERC before the end of the study (i.e., day 25 [paclitaxel] or 29 [AC]) and samples with a CT standard deviation > 1 (run in duplicate) were excluded from analysis. Total composite z-scores were calculated for cytokine analysis to determine an overall separate inflammatory profile for the cortex and hypothalamus. Z-scores were calculated by subtracting the mean relative mRNA expression level from that of each sample and dividing by the standard deviation (e.g., [sample-mean]/SD). Z-scores for IL-1β, IL-6, and TNF-α were then added to get a composite z-score representing the inflammatory profile in either the cortex or hypothalamus (Zhang et al., 2023). Outliers in relative expression values were determined *a priori* via Grubb's test and excluded from the mean and SD calculations, and all samples from the corresponding animal were excluded from analysis. Animals with one or more excluded cytokine(s) due to a CT standard deviation > 1 between technical replicates were excluded from composite z-score analysis (D’Aprile et al., 2025).

### 2.8 Serum analysis

#### 2.8.1 Cytokine assay

Blood was centrifuged for 25 min at 2500 g at 4 °C to separate serum. A V-PLEX Proinflammatory Panel 1 (mouse) Kit (Meso Scale Diagnostics) was used to measure serum levels of IFN-γ, IL-1β, IL-2, IL-4, IL-5, IL-6, IL-10, IL-12p70, KC/GRO, and TNF-α per the manufacturer's instructions.

#### 2.8.2 Circulating cytokine analysis

Samples from mice that reached ERC before the end of the study (i.e., day 25 [paclitaxel] or 29 [AC]) were excluded from analysis. Raw cytokine concentrations (pg/mL) were log-transformed to standardize values and reduce skew (Varadhan et al., 2013). Total composite z-scores were calculated for cytokine concentrations to determine overall pro-inflammatory (IL-1β, IL-6, TNF-α, IFN-γ, IL-2, and CXCL1) and anti-inflammatory (IL-10 and IL-4) profiles for each animal. Of note, IL-2 was included in the pro-inflammatory profile due to its pro-inflammatory properties associated with CRF (Bower, 2007; Schubert et al., 2007; Li et al., 2025). IL-4 concentrations for paclitaxel-treated mice were below the curve fit range for the assay and were not included in analysis. Z-scores were calculated as described above. Outliers in log-transformed values were determined via Grubb's test and excluded from the mean and SD calculations, and all samples from the corresponding animal were excluded from analysis (D’Aprile et al., 2025).

### 2.9 Statistical analysis

Outliers were determined *a priori* via the Grubb's Test and removed from analysis. Mixed-effects analyses were used to analyze change in measurements throughout the study (body mass, tumor volume, wheel counts, number of bouts, and avg. time/bout) followed by Fisher's LSD. Direct comparisons between groups receiving chemotherapy at different times (terminal tumor mass, AUCs, and relative expression of cytokines) were compared using two-tailed unpaired student's t-tests. P ≤ 0.05 was considered significant for all analyses. All graphs represent mean with error bars representing SEM. Statistics were run using GraphPad Prism 10.

## 3 Results

### 3.1 Mice receiving paclitaxel at ZT6 demonstrated increased VWRA

**Fig. 1:**
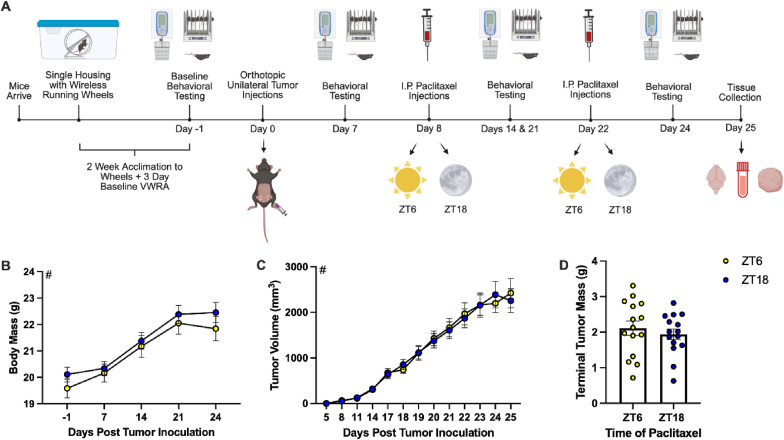
**Time of paclitaxel administration does not affect tumor progression.** (A) Experimental timeline. Biorender.com (B) Body mass over time. (C) Tumor volume over time. (D) Tumor mass at the time of tissue collection. Significant effect of # day post tumor inoculation. Data are presented as mean ± SEM. (B,C) n = 13-15/ZT/day, (D) n = 15/ZT.

To determine if timed administration of paclitaxel affects fatigue-like behavior (FLB), mice bearing primary EO771 mammary tumors were injected intraperitoneally with paclitaxel at either *zeitgeber* time (ZT) 6 (mid-inactive phase) or ZT18 (mid-active phase; Fig. 1A). Timed administration of paclitaxel did not significantly affect body mass (Fig. 1B) or tumor volume (Fig. 1C) throughout the study. Terminal tumor mass (tumor mass at the time of tissue collection) also did not differ based on time of paclitaxel administration (Fig. 1D).

**Fig. 2:**
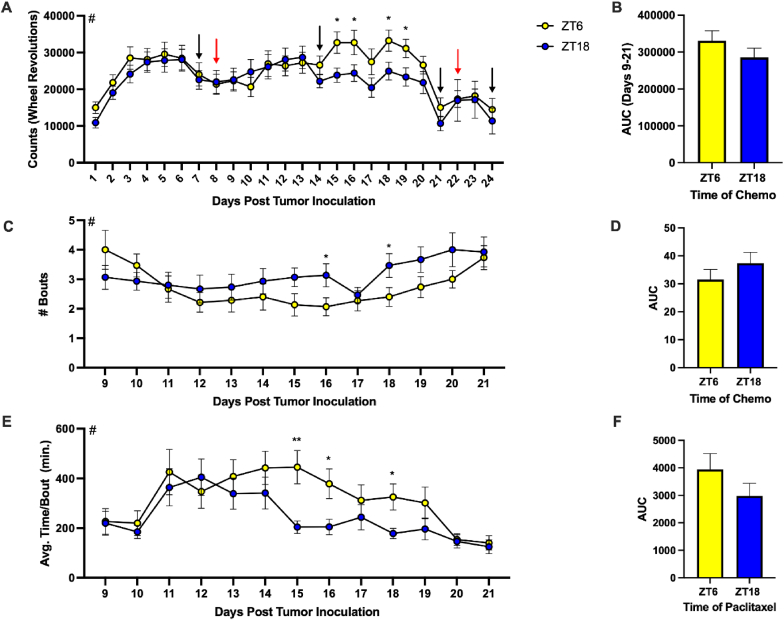
**Mice receiving paclitaxel at ZT6 demonstrated increased VWRA.** (A) Total daily counts (wheel revolutions) during the active phase (lights off; ZT12-ZT23) throughout the study. Red arrows indicate days of chemotherapy injection and black arrows indicate days of behavioral testing. (B) AUC of counts on days 9-21 post tumor inoculation (PTI). (C) Number of daily activity bouts during the active phase days 9-21 PTI. (D) AUC of *C*. (E) Average daily time per activity bout days 9-21 PTI. (F) AUC of *E*. Data are presented as mean ± SEM. Significant effect of # day post tumor inoculation, ∗p ≤ 0.05, ∗∗p ≤ 0.01. (A) n = 7-15/ZT/day due to a technical error preventing wheel data collection following day 21 in some mice, (B,D,F) n = 176-183, (C,E) n = 14-15/ZT/day. (For interpretation of the references to color in this figure legend, the reader is referred to the Web version of this article.)

Next, we sought to assess fatigue-like behavior in tumor-bearing mice. Although not evident from behavioral tests commonly used to assess physical FLB (grip strength [Takeshita et al., 2017] and wire hang [Ray et al., 2011]; Fig. S1), a fatigue-like phenotype was demonstrated by the reduction in VWRA (Fig. S2). There was a main effect of day when assessing daily counts (i.e., wheel revolutions) during the active phase (Fig. 2A; F_(4.180,107.2)_ = 11.79, p < 0.0001). Specifically, daily counts visibly delineated during the week leading up to the second dose of paclitaxel (Fig. 2A). Because of this and the drop-off of activity following the second dose (∼40 percent reduction from 3 days prior to first chemotherapy injection), further analysis focused on the 14-day period between the two doses of paclitaxel (red arrows). Although the representation of the total activity (area under the curve; AUC) during this time period was not significantly different between mice injected at either time (Fig. 2B), counts from mice treated at ZT6 on days 15, 16, 18, and 19 post tumor inoculation were significantly increased relative to ZT18 treated mice (Fig. 2A), indicating a reduction of FLB. Similar effects were also observed in bout numbers and average bout length. Indeed, there was a main effect of day post tumor inoculation in both the number of bouts (Fig. 2C; F_(6.803, 187.7)_ = 3.616, p = 0.0013) and time per bout (Fig. 2E; F_(4.942, 134.7)_ = 7.050, p < 0.0001). However, the cumulative number of attempted bouts and average bout length between the two doses of paclitaxel were not significant (Fig. 2D and F). Mice treated at ZT18 demonstrated significantly higher numbers of attempted bouts of activity on days 16 and 18 post tumor inoculation (PTI; Fig. 2C). Additionally, the average time spent running per bout was significantly lower on these days (Fig. 2E) as well as day 15 PTI, indicating increased FLB. Together, these data demonstrate a reduction of FLB in mice treated at ZT6 compared to mice treated at ZT18.

### 3.2 Inflammatory profiles are elevated in the hypothalamus of mice treated with paclitaxel at ZT18

**Fig. 3:**
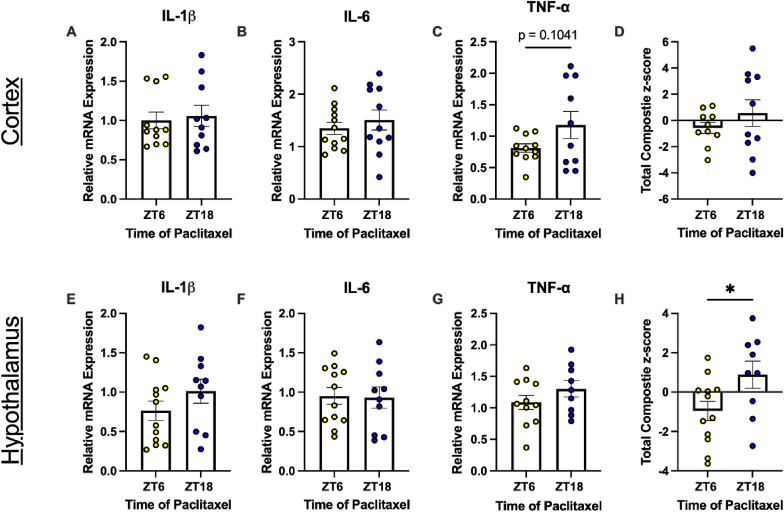
Inflammatory profiles are elevated in the hypothalamus of mice treated with paclitaxel at ZT18. Relative mRNA expression of cortical (A) IL-1β, (B) IL-6, and (C) TNF-α. (D) Total inflammatory composite z-score of relative mRNA expression in the cortex. Relative mRNA expression of hypothalamic (E) IL-1β, (F) IL-6, and (G) TNF-α. (H) Total inflammatory composite z-score of relative mRNA expression in the hypothalamus. Data are presented as mean ± SEM. n = 10-12/ZT.

One prevalent mechanistic explanation is the cytokine hypothesis of CRF, which asserts that pro-inflammatory cytokines (specifically, IL-1β, IL-6 and TNF-α) trigger a central response that leads to fatigue (Bower, 2014). Indeed, these cytokines have been correlated with CRF in clinical studies (Bower, 2014; Meyers et al., 2005). To test this hypothesis in our model, we measured relative mRNA expression of IL-1β, IL-6, and TNF-α in the cortex and hypothalamus of tumor-bearing mice following the second dose of paclitaxel. There were no time-of-treatment differences in relative mRNA levels of individual cytokines in either the cortex or hypothalamus (Fig. 3, Table 1). The total inflammatory composite z-scores were not significantly different in the cortex (Fig. 3D; Table 1). However, inflammatory composite z-scores were significantly increased in the hypothalamus of mice injected with paclitaxel at ZT18 relative to ZT6 (Fig. 3H,Table 1), suggesting an elevated inflammatory response in this region. We also conducted a multiplex ELISA to analyze concentrations of ten circulating inflammatory factors and again observed no time-of-treatment differences in concentrations (with the exception of elevated CXCL1 [KC/GRO] at ZT6; Fig. S3, Table 2), indicating that changes to the circulating cytokine profile observable three days following the second dose of chemotherapy likely does not contribute to FLB demonstrated between doses. However, given the elevated inflammatory profile in the hypothalamus of these mice, this could be reminiscent of a region-specific central inflammatory mechanism driving increased FLB in mice treated at ZT18.

**Table 1:** Relative mRNA expression of central cytokines following second paclitaxel administration.

Cytokine	Location	ZT6 Mean ± SEM	ZT18 Mean ± SEM	p-value
IL-1β	Cortex	1.002 ± 0.1058	1.059 ± 0.1354	0.7416
IL-6	Cortex	1.351 ± 0.1161	1.511 ± 0.1916	0.4747
TNF-α	Cortex	0.8108 ± 0.0667	1.179 ± 0.2145	0.1041
Total Composite z-score	Cortex	−0.5659 ± 0.4179	0.5659 ± 1.014	0.3159
IL-1β	Hypothalamus	0.7634 ± 0.1222	0.9845 ± 0.1406	0.2466
IL-6	Hypothalamus	0.9515 ± 0.1061	0.8999 ± 0.1251	0.7549
TNF-α	Hypothalamus	1.088 ± 0.1116	1.211 ± 0.1474	0.5112
Total Composite z-score	Hypothalamus	−0.9590 ± 0.4921	0.8877 ± 0.6896	0.0370

**Table 2:** Circulating concentrations of inflammatory markers following second paclitaxel administration.

Cytokine	ZT6 Mean (pg/mL) ± SEM	ZT18 Mean (pg/mL) ± SEM	p-value
IL-1β	3.721 ± 0.7740	3.862 ± 0.8089	0.9014
IL-6	81.36 ± 17.05	56.40 ± 13.18	0.2603
TNF-α	14.97 ± 1.154	15.90 ± 1.302	0.6007
IFN-γ	0.6537 ± 0.0832	0.5451 ± 0.0586	0.3061
IL-2	0.6875 ± 0.0182	0.5853 ± 0.0556	0.0849
KC/GRO	854.8 ± 133.0	487.5 ± 42.42	0.0247
IL-5	5.217 ± 0.6547	4.804 ± 0.5179	0.6366
Total Pro-Inflammatory Composite z-score	0.9983 ± 1.218	−1.089 ± 1.331	0.9983
IL-10	12.90 ± 1.040	11.89 ± 1.696	0.6067
IL-4	NA	NA	
Total Anti-Inflammatory Composite z-score	0.0535 ± 0.1782	−0.0584 ± 0.4016	0.7955
IL-12p70	NA	NA	

NA-below fit curve range of assay.

### 3.3 Response to AC affects tumor progression

**Fig. 4:**
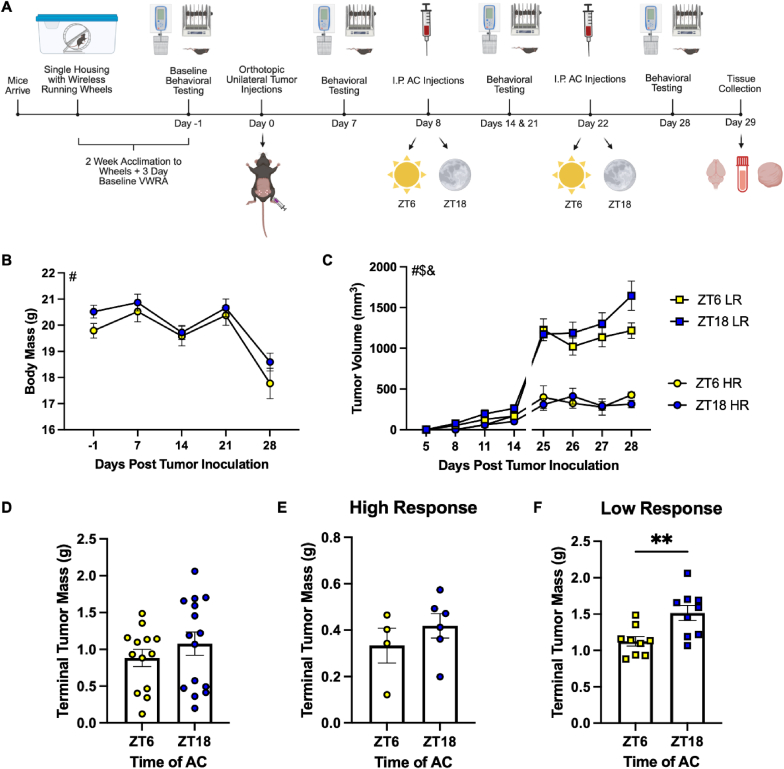
**Response to AC affects tumor progression.** (A) Experimental timeline. Biorender.com (B) Body mass over time. (C) Tumor volume over time separated by a high response (HR) and low response (LR) to AC. Terminal Tumor volume in (D) all mice, (E) mice with HR, and (F) mice with LR to AC. Data are presented as mean ± SEM. Significant effect of # day post tumor inoculation, $ group (i.e., ZT6 LR, ZT18 LR, ZT6 HR, ZT18 HR), & interaction, ∗∗p ≤ 0.01. (B) n = 8-15/ZT/day (variable due to some mice reaching ERC before day 28), (C) n = 2-6/ZT/day for HR, n = 6-9/ZT/day for LR (variable due to some mice reaching ERC before day 28), (D) n = 13-15/ZT, (E) n = 4-6/ZT, (F) n = 9/ZT.

Using the same model, we next sought to determine the effect of timed administration of a second chemotherapeutic, AC, on FLB (Fig. 4A). As with the paclitaxel, body mass of tumor-bearing mice did not differ significantly based on time of treatment (Fig. 4B). However, there were two distinctly different clusters based on volume (Fig. 4C) and terminal tumor mass (Fig. 4D). These two significantly different groups were separated based on the geometric mean of terminal tumor mass (0.7404 g for ZT6 mice and 0.8773 g for ZT18 mice) and volume (912.2 mm^3^ for ZT6 mice and 890.5 mm^3^ for ZT18 mice; Fig. S4). The geometric mean is commonly used in clinical studies (specifically in breast cancer [Kuroishi et al., 1990]) to assess volume and progression due to the logarithmic growth of tumors (Demidenko, 2010). Mice were then split into two groups based on if their terminal tumor masses and volumes were above or below the geometric mean of all values within a timepoint (i.e., ZT6 or ZT18) for further analysis (Fig. S4). Those below the geometric mean are denoted as having a relative “high response” (Fig. 4E), and those below are denoted as having a relative “low response” (Fig. 4F). Interestingly, terminal tumor masses were significantly reduced in mice that had a relative low response to AC therapy and were treated at ZT6 (1.125 ± 0.0664 g) compared to ZT18 (1.516 ± 0.1047 g) (Fig. 4F; t = 3.148. p = 0.0062), indicating a potential advantage of a ZT6 treatment time unrelated to FLB.

### 3.4 Mice treated at ZT6 with a relative high response to AC demonstrated significantly reduced fatigue-like behavior

**Fig. 5:**
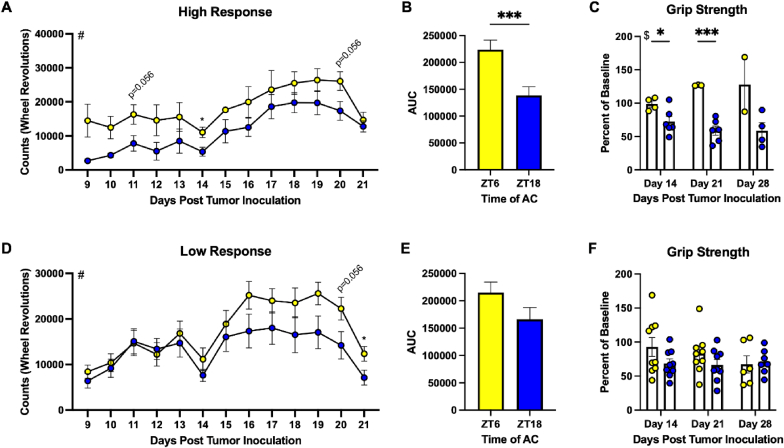
Mice treated at ZT6 with a relative high response to AC demonstrated significantly reduced fatigue-like behavior. Number of daily activity bouts during the active phase days 9-21 PTI in (A) high and (D) low response mice. (B) AUC of *A*. (E) AUC of *D*. Normalized grip strength (body mass/grip strength) on day 7 (prior to chemotherapy) was used as a baseline to calculate normalized grip strength as a percentage of baseline throughout the study in mice with (C) high response and (F) low response to AC. Data are presented as mean ± SEM. Significant effect of # day post tumor inoculation and $ time of treatment, ∗p ≤ 0.05, ∗∗∗p ≤ 0.001. (A) n = 3-6/ZT/day, (C) n = 2-6/ZT/day (variable due to some mice reaching ERC before day 28), (D) n = 8-9/ZT/day, (F) n = 6-9/ZT/day.

**Fig. 6:**
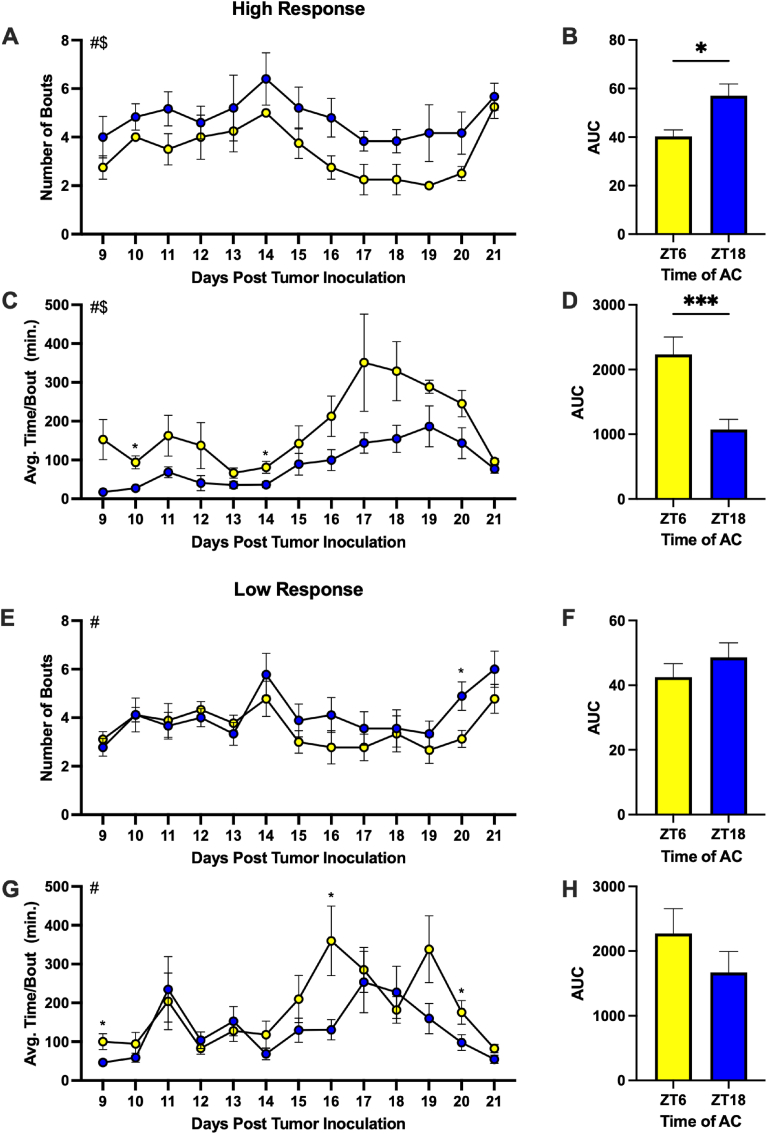
**Mice treated at ZT18 with a relative high response to AC demonstrated an increased number of bouts of activity that were shorter in duration.** Number of daily activity bouts during the active phase days 9-21 PTI in mice with (A) high response and (E) low response. Average daily time per activity bout days 9-21 PTI in mice with (C) high response and (G) low response. (B) AUC of *A*, (D) *C*, (F) *E*, and (H) *G*. Data are presented as mean ± SEM. Significant effect of # day post tumor inoculation and $ time of AC injection, ∗p ≤ 0.05, ∗∗∗p ≤ 0.001. (A,C) n = 3-6/ZT/day, (E,G) n = 8-9/ZT/day.

Similar to mice treated with paclitaxel, mice treated with AC demonstrated clear FLB through VWRA. As observed previously, there was also a substantial drop-off of activity following the second dose of chemotherapy (i.e., ∼90 percent reduction relative to baseline), leading us to continue analysis in time-of-treatment differences in the two-week period between doses (Fig. S5). Mice with a high response to AC and treated at ZT6 demonstrated significantly elevated counts throughout this 14-day period (Fig. 5A and B; t = 3.414, p = 0.0009), resulting in higher wheel counts on days 11 (p = 0.056), 14 (p < 0.05) and 20 (p = 0.056). Interestingly, there was a significant effect of time of treatment in the grip strength of mice with a high response to AC (Fig. 5C; F_(1, 19)_ = 39.96, p < 0.0001). When compared to a baseline of their own grip strength prior to treatment, high responders treated at ZT6 had elevated grip strengths relative to those treated at ZT18 on days 14 and 21 post tumor inoculation (PTI) (Fig. 5C). However, there were no significant differences in latency to fall in the wire hang task (Fig. S6). These results are in contrast to mice with a relative low response to AC. Mice with a low response to AC and treated at ZT6 demonstrated elevated activity only the week prior to the second dose and no significant differences in cumulative activity or grip strength (Fig. 5D–F). High responding mice treated with AC at ZT18 demonstrated an increased number of bouts of activity (Fig. 6A [F_(1, 8)_ = 3.144, p = 0.0322] & B [t = 2.572, p = 0.0116]) that were shorter in duration (Fig. 6C [F_(1, 8)_ = 9.956, p = 0.0135] & D [t = 3.976, p = 0.0001]) than those treated at ZT6, indicating increased FLB. Although cumulative number of bouts (Fig. 6F) and average time per bout (Fig. 6H) were not significant in low responders, mice treated at ZT18 demonstrated a higher number of bouts on day 20 and decreased time per bout on days 16 and 20 (Fig. 6E and G). Overall, these data represent prominent time-of-treatment differences in FLB that are exacerbated by a higher response to AC.

### 3.5 Relative mRNA expression of central inflammatory markers following the second dose of AC are not affected by time of chemotherapy administration

**Fig. 7:**
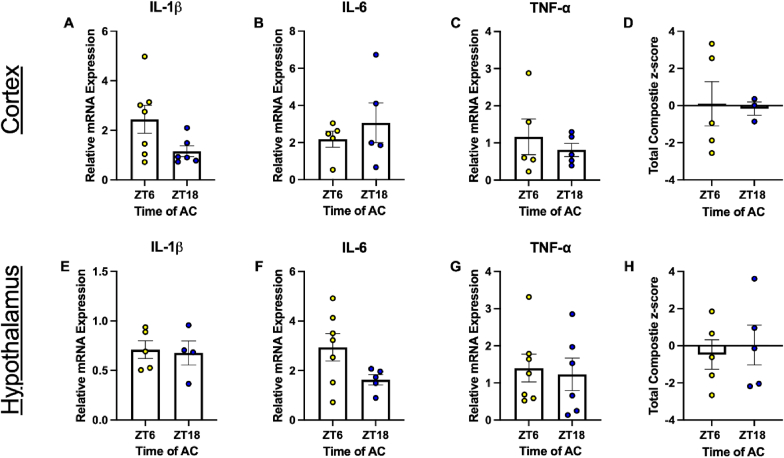
Relative mRNA expression of central inflammatory markers following the second dose of AC is not affected by time of chemotherapy administration in mice with low response to AC. Relative mRNA expression of cortical (A) IL-1β, (B) IL-6, and (C) TNF-α in mice with low response to AC. (D) Total inflammatory composite z-score of relative mRNA expression in the cortex. Relative mRNA expression of hypothalamic (E) IL-1β, (F) IL-6, and (G) TNF-α in mice with low response to AC. (H) Total inflammatory composite z-score of relative mRNA expression in the hypothalamus. Data are presented as mean ± SEM. n = 4-7/ZT.

We again examined both central and peripheral inflammatory markers. Due to the number of mice that reached the end of the study (i.e., the starting n was lower in HR, and mice injected at both timepoints reached chemo toxicity-related ERC at an equal rate), we were only able to statistically compare relative expression of cytokines in mice with a low response to AC (Fig. 7; Table 3). However, comparison of high responders is illustrated in (Fig. S7). There were no significant differences between time of treatment in expression of IL-1β, IL-6, or TNF-α in either the cortex or hypothalamus (Fig. 7; Table 3) of these mice, nor were there differences in circulating levels of pro-inflammatory markers (Fig. S8; Table 4) when response groups were combined (due to the restricted number of serum samples obtained in mice treated with AC). There were also no differences between composite inflammatory profiles in the cortex (Fig. 7D; Table 3), hypothalamus (Fig. 7H; Table 3), or blood (Fig. S8; Table 4) of these mice.

**Table 3:** Relative mRNA expression of central cytokines in mice with low response following second AC administration.

Cytokine	Location	ZT6 Mean ± SEM	ZT18 Mean ± SEM	p-value
IL-1β	Cortex	2.444 ± 0.5614	1.154 ± 0.2187	0.0699
IL-6	Cortex	2.186 ± 0.4334	3.068 ± 1.069	0.4665
TNF-α	Cortex	1.163 ± 0.4831	0.8108 ± 0.1790	0.5130
Total Composite z-score	Cortex	0.0991 ± 1.194	−0.1652 ± 0.3602	0.8752
IL-1β	Hypothalamus	0.7097 ± 0.0895	0.6773 ± 0.1211	0.8318
IL-6	Hypothalamus	2.935 ± 0.5531	1.629 ± 0.2095	0.0860
TNF-α	Hypothalamus	1.398 ± 0.3727	1.232 ± 0.4380	0.7765
Total Composite z-score	Hypothalamus	−0.4748 ± 0.7978	0.0400 ± 1.070	0.7097

**Table 4:** Circulating concentrations of inflammatory markers in all mice following second AC administration.

Cytokine	ZT6 Mean (pg/mL) ± SEM	ZT18 Mean (pg/mL) ± SEM	p-value
IL-1β	3.649 ± 1.174	5.041 ± 0.8646	0.4027
IL-6	25.67 ± 2.574	61.56 ± 12.34	0.1514
TNF-α	14.04 ± 1.176	20.07 ± 2.544	0.1908
IFN-γ	0.4853 ± 0.1501	0.8448 ± 0.1922	0.3058
IL-2	0.9093 ± 0.1954	1.099 ± 0.1346	0.4677
KC/GRO	104.6 ± 2.741	213.9 ± 37.12	0.1466
IL-5	4.359 ± 1.313	3.702 ± 0.7963	0.6706
Total Pro-Inflammatory Composite z-score	−2.664 ± 1.211	1.065 ± 1.361	0.1336
IL-10	10.71 ± 2.323	12.83 ± 1.371	0.4417
IL-4	0.5777 ± 0.5394	0.8003 ± 0.1962	0.6328
Total Anti-Inflammatory Composite z-score	−0.3892 ± 0.7304	0.5137 ± 0.3873	0.2894
IL-12p70	NA	NA	

NA-below fit curve range of assay.

## 4 Discussion

Cancer-related fatigue is the most commonly reported adverse event related to anti-tumor therapy. Specifically, >80% of people receiving outpatient chemotherapy reported some level of CRF in a study with over 400 participants (Hofman et al., 2007). Although not specific to one cancer type, CRF is especially prevalent in breast cancer (Kang et al., 2023), and can last up to a decade or longer after completion of treatment in >30% of breast cancer survivors (Bower et al., 2006). We therefore sought to determine if chronomodulation of components of dose-dense AC therapy (i.e., AC and paclitaxel), an effective neoadjuvant strategy for early-stage triple negative breast cancer (Bonadio et al., 2024), could reduce CRF. Using a murine model of primary triple negative breast cancer (TNBC), we demonstrated that there is a time-of-treatment effect on fatigue-like behavior, particularly following the first chemotherapy injection.

We first demonstrated that administration of paclitaxel at either the mid-inactive phase (ZT6) or mid-active phase (ZT18) did not alter tumor progression (Fig. 1). However, the same was not true for the administration of AC (Fig. 4). In mice with a relative low response (i.e., terminal tumor mass and volume above the geometric mean) to AC, injection at ZT6 significantly reduced tumor mass at the time of tissue collection (Fig. 4F). This is unsurprising, as it is not uncommon for treatment-naïve TNBC to have resistance to neoadjuvant AC, resulting in substantial tumor burden following treatment and before surgical resection (Echeverria et al., 2019). Indeed, it was determined that AC administration in a PDX model of TNBC did not completely halt tumor progression, but rather slowed or regressed tumor growth acutely after treatment, but not permanently (Echeverria et al., 2019). The same study also identified that paclitaxel alone did not halt tumor progression (Echeverria et al., 2019). This aligns with the current study, as the experimental timeline using AC (Fig. 4A) was longer than that using paclitaxel (Fig. 1A), which was more highly restricted by ERC (20 mm tumor). Transient mechanisms of sensitivity to AC include histological features and differences in the transcriptome/proteome (compared to AC-sensitive tumors) (Echeverria et al., 2019). Given that ∼43% of mouse protein-coding genes oscillate based on time of day (Zhang et al., 2014) (of which, up to 16% are involved in the cell cycle [Miller et al., 2007]), it is a potential that specific timing of AC administration confers an advantage in the altered genetic architecture of AC-resistant tumors. However, given that the same tumor cells were injected into each mouse in the current study, it is more likely an individual-mediated mechanism. More work is needed to investigate the mechanism driving reduced tumor burden at ZT6 (in low responders to AC), as this may be a promising finding for the treatment of patients with neoadjuvant AC-resistant TNBC.

Mice treated with paclitaxel at ZT6 demonstrated significantly higher wheel-running activity on four of the seven days (15, 16, 18, and 19 days PTI) leading up to the second dose of chemotherapy (Fig. 2A). On two of these days (16 and 18), mice treated at ZT18 had a significantly increased total number of activity bouts (Fig. 2C) which were significantly shorter than those of mice treated at ZT6 (Fig. 2E). These data resemble wheel-running activity observed in mice with a relative low response to AC. In both cases, mice treated at ZT6 demonstrated increased wheel-running activity later (relative to mice with high response to AC) following the first dose of chemotherapy (i.e., around days 14-16 rather than day 9). Together, these data demonstrate reduced FLB at ZT6 and potentially indicate an effect on a more chronic fatigue-like phenotype that is susceptible to chronomodulation, which should be examined in future studies.

Mice treated at ZT6 with a relative high response to AC demonstrated significantly increased wheel-running activity compared to their ZT18 counterparts as evidenced by higher active wheel counts (Fig. 5A–B) and fewer bouts of activity that were longer in duration (Fig. 6A–D). These mice also demonstrated a higher grip strength relative to baseline (Fig. 5C). Together, these data demonstrate an enhanced reduction of FLB in mice with a high response when AC was administered at ZT6 relative to ZT18. This also indicates that sensitivity to chronomodulation may be positively correlated to the level of responsiveness to the drugs.

One possible explanation for time-of-treatment differences in CRF observed throughout the study is circadian regulation of blood-brain-barrier (BBB) permeability (Pulido et al., 2020; Zhang et al., 2021; Zhang et al., 2018). Although limited, research suggests time-of-day differences in permeability are due to oscillations in efflux transport of xenobiotics (e.g., chemotherapeutics) at the BBB (Pulido et al., 2020; Zhang et al., 2021; Zhang et al., 2018). Indeed, previous studies have demonstrated that there are time-of-treatment differences in intratumoral ^14^C-paclitaxel concentrations in a mouse model of breast cancer brain metastases, with higher levels of drug reaching the brain during the active phase (Walker II et al., 2021). Further, one study demonstrates that efflux-mediated transport at the BBB is driven by neuronal activity. Specifically, transcripts of efflux transporters were downregulated when cortical neurons were activated, thus decreasing efflux activity and increasing BBB permeability (Pulido et al., 2020). These studies align with the present findings, as it is reasonable to suggest that higher concentrations of chemotherapeutics are crossing the BBB when administered at ZT18 (i.e., the active phase) to invoke a centrally mediated fatigue-like phenotype.

Voluntary wheel-running activity was used as the main indicator of FLB. Although other behavioral paradigms (i.e., grip strength meter [Zhu et al., 2019] and wire hang test [Ray et al., 2011]) were also used, VWRA is more sensitive to FLB and provides a near constant measurement, unlike behavioral tests that are performed periodically. Indeed, it is not uncommon in models of chemotherapy-related fatigue to demonstrate FLB via VWRA but not behavioral tasks (Zombeck et al., 2013; Phan et al., 2024). CRF is especially hard to model, as this is something that heavily relies on self-reporting in clinic, which cannot be recapitulated using animals (Zombeck et al., 2013). Because there are different components of fatigue (i.e., motivational and physical) (Dantzer et al., 2025), it is debated which one of these VWRA represents. Due to the obvious physicality of wheel-running, some researchers argue that this a measurement of physical fatigue. In a murine study of chemoradiotherapy-induced fatigue, the authors concluded that treatment induced physical fatigue via decreased VWRA, but not motivational fatigue as evidenced by a lack of deviation from the control group in performance in reward-based tasks (Phan et al., 2024). However, others argue that because wheel-running is voluntary, it is a motivational behavior. Indeed, mice can be trained to unlock running wheels via nose poke, indicating a motivation to run (Muguruza et al., 2019; Zhang et al., 2022). Further, VWRA has been demonstrated to increase c-Fos (a neuronal activation marker) staining in the medial prefrontal cortex, nucleus accumbens, and dorsal striatum, all of which are involved in motivational networks (Zhang et al., 2022). It is highly likely that CRF is a combination of motivational and physical fatigue driven by complex physiological networks of both components that interact with each other. Indeed, patients experiencing CRF exhibit altered connections in neural pathways driving effort-based decision making (André et al., 2021) that could impact motivation to engage in physical activity. Physical activity has been demonstrated to alleviate CRF, particularly in breast cancer survivors (Cramp and Byron-Daniel, 2012), thus demonstrating a complicated relationship between physical and motivational components of CRF. Here we demonstrate reduced VWRA in mice treated at ZT18 with or without increased physical FLB (i.e., grip strength/wire hang), potentially indicating motivational FLB in the presence (i.e., mice treated at ZT6 with high response to AC) or absence (i.e., mice treated with paclitaxel and those with a low response to AC) of physical FLB. This could potentially be attributed to the severity of FLB, as mice with high response to AC treated at ZT18 demonstrated consistently decreased VWRA (relative to mice treated at ZT6) throughout the two-week period between doses of chemotherapy, which was accompanied by a significant decrease in grip strength relative to their ZT6 counterparts. Conversely, mice treated at ZT6 with paclitaxel or that had a low response to AC only demonstrated significantly reduced FLB on some days during the second week following the first dose, potentially indicating a more moderate FLB that skews more toward a motivational component without an apparent lack of physical capability.

Lastly, we sought to examine the cytokine hypothesis of CRF. There were no significant differences in expression of cortical or hypothalamic IL-1β, IL-6, or TNF-α based on time of treatment using either chemotherapeutic (Fig. 3, Fig. 7; Table 1, Table 3). However, mice treated with paclitaxel at ZT6 had a reduced inflammatory profile in the hypothalamus compared to mice treated at ZT18 (Fig. 3H). These regions were examined due to their roles in fatigue and related behavior. The cortex, specifically the prefrontal cortex, is responsible for decision-making and motivation (Soutschek and Tobler, 2020), and upregulated transcripts in inflammatory pathways have been observed alongside FLB in mice (Azzinnari et al., 2014). The hypothalamus houses the suprachiasmatic nucleus, which is responsible for synchronization of circadian rhythms throughout the body. Indeed, activity rhythms can be dysregulated by doxorubicin-induced CRF (Wang et al., 2024). Further, a chemotherapeutic cocktail including cyclophosphamide has been demonstrated to induce FLB by decreasing murine hypothalamic orexin activity (responsible for arousal/wakefulness). This was accompanied by increased hypothalamic IL-6 and TNF-α transcript levels six to 24 h following treatment (Weymann et al., 2014). Indeed, this provides further evidence that the presently observed increased inflammation in the hypothalamus of paclitaxel-treated mice (ZT18) could be contributing to the corresponding heightened fatigue-like behavior. However, lack of an inflammatory phenotype in AC treated mice in the present study does not definitively rule out TOD differences in cytokine induced FLB. Further, this does not mean that the same was not true in AC-treated mice. Indeed, studies have previously demonstrated that hypothalamic and hippocampal IL-1β, TNF-α, and other inflammatory marker transcripts are increased when AC is administered during the active phase but not the inactive phase (Borniger et al., 2017). It is unsurprising that we did not observe time-of-treatment differences in hypothalamic inflammation in AC-treated mice due to our experimental timeline. Tissue was collected in mice treated with AC seven days after the second dose (21 days following the first dose; Fig. 4A) following a large reduction of wheel-running activity in both treatment groups (Fig. S5). However, time-of-treatment differences in FLB were observed mainly prior to the second dose of both chemotherapeutics. We therefore cannot conclude that the mechanism driving differences in FLB in AC-treated mice is or is not related to central inflammation based on the current study. Future studies should assess the central inflammatory profile of mice treated at different times of day following the first dose when differences in FLB are apparent.

The present study was limited primarily by the length of the experimental timelines. Based on previous studies, IL-6, TNF-α, and IL-1β cytokine levels peak seven days after chemotherapy injection within the brain (Walker II et al., 2020). We therefore sought to collect tissue seven days following the last chemotherapy dose. However, due to the rapid growth of EO771 tumors and limited responsiveness to chemotherapy, we were only able to administer two doses and were unable to wait seven days after the second dose to collect tissue when using paclitaxel. Future studies should assess chronomodulation in slower-growing models, including patient-derived tumors. Although one retrospective study observed an increase in patients receiving neoadjuvant chemotherapy for triple negative breast cancer (45% to 89% from 2015 to 2020, respectively [Rosińska et al., 2024]), studies similar to this one should investigate FLB in adjuvant systemic treatment. This would allow for a longer study to determine how timed administration of chemotherapy affects chronic FLB/CRF as well as add to the growing body of translational data on chronomodulation in cancer treatment. Future studies could also consider examining how these drugs impact circadian and activity rhythms. Indeed, paclitaxel has been demonstrated to dampen rhythms of clock genes in the SCN in mice kept under constant darkness (Tapp et al., 2025). Further, mice treated with doxorubicin demonstrated significantly disrupted activity cycles compared to vehicle-treated mice under constant dark conditions. However, these disruptions were insignificant between doxorubicin- and vehicle-treated mice when entrained to a 12:12 light/dark cycle (Wang et al., 2022). Mice in the present study were similarly entrained to a light/dark cycle. Because circadian rhythms by definition must persist in constant conditions (Aschoff, 1979), the present study is not designed to analyze the potential effects of these chemotherapies on circadian rhythms. Therefore, further studies conducted in constant dark conditions are needed to determine the effects of these drugs on circadian rhythms and VWRA.

Inflammatory analysis was limited by inter-sample variability. Although outliers were removed from analysis, there is still a moderate SEM of relative expression levels of cytokines within treatment groups (Fig. 3, Fig. 7). This could be due to a low n (i.e., from some animals reaching ERC, which were excluded from tissue analysis). Future studies should also analyze inflammation in other motivation-associated regions such as the nucleus accumbens and dorsal striatum to provide a fuller picture of dysregulation. Lastly, as discussed above, clinically CRF is highly dependent on self-reports in patients, making it challenging to assess in animal models. Adding to this challenge is the differentiation between physical and motivational fatigue. Future studies could assess motivation-specific tasks like the effort-based forage task (Grayson et al., 2025), the weighted door task (Wells et al., 2026), or the ladder task (Wells et al., 2026) with the caveat that these tests can be confounded by depressive-like behavior and physical fatigue. However, VWRA is generally agreed to be a reliable indication of FLB (Zombeck et al., 2013; Wolff et al., 2018). Notably, the present data indicate that time of treatment can affect fatigue-like behavior. Therefore, these data support expanding preclinical and clinical research to determine how chronomodulated administration of existing therapies may improve quality of life in breast cancer survivors.

## CRediT authorship contribution statement

**Claire O. Kisamore:** Conceptualization, Data curation, Formal analysis, Investigation, Methodology, Project administration, Visualization, Writing – original draft, Writing – review & editing. **Caleb A. Kisamore:** Formal analysis, Investigation, Methodology. **Jayla M. Boyd:** Investigation, Methodology. **Paul J. Owolabi:** Investigation. **Divya B. Kadri:** Investigation. **Alan D. Mizener:** Data curation, Formal analysis, Investigation, Software, Writing – review & editing. **Emidio E. Pistilli:** Conceptualization, Funding acquisition, Methodology, Project administration, Resources, Supervision, Writing – review & editing. **William H. Walker:** Conceptualization, Funding acquisition, Methodology, Project administration, Resources, Supervision, Writing – original draft, Writing – review & editing.

## Declaration of competing interest

The authors declare that they have no known competing financial interests or personal relationships that could have appeared to influence the work reported in this paper.

## Data Availability

Data will be made available on request.

## Glossary

AC: Adriamycin (doxorubicin) & cyclophosphamide
AUC: area under the curve
CRF: cancer-related fatigue
ERC: early removal criteria
FLB: fatigue-like behavior
HR: high response
ipRGC: intrinsically photosensitive retinal ganglion cell
LR: low response
PTI: post tumor inoculation
SCN: suprachiasmatic nucleus
VWRA: voluntary wheel-running activity
ZT: *Zeitgeber* time
