# Supplementary material for: Chronotherapeutic administration of neoadjuvant chemotherapy reduces cancer-related fatigue-like behavior
Source: Brain Behav Immun Health. 2026 May 19;54:101262. doi: 10.1016/j.bbih.2026.101262 (PMC13226266; doi:10.1016/j.bbih.2026.101262)
Supplement: Multimedia component 1 [file mmc1.docx]

**Supplementary Figures**

**Figure S1 There were no behavioral differences in ZT6 versus ZT18 paclitaxel administration.** Mice underwent once-weekly (A) grip strength (B) wire hang, and (C) rotarod tests. Data are represented as percentage of baseline, which was tested the day prior to tumor inoculation. Data are presented as mean ± SEM. Significant effect of $ time of treatment; no group differences observed. n=12-15/time/day.

**
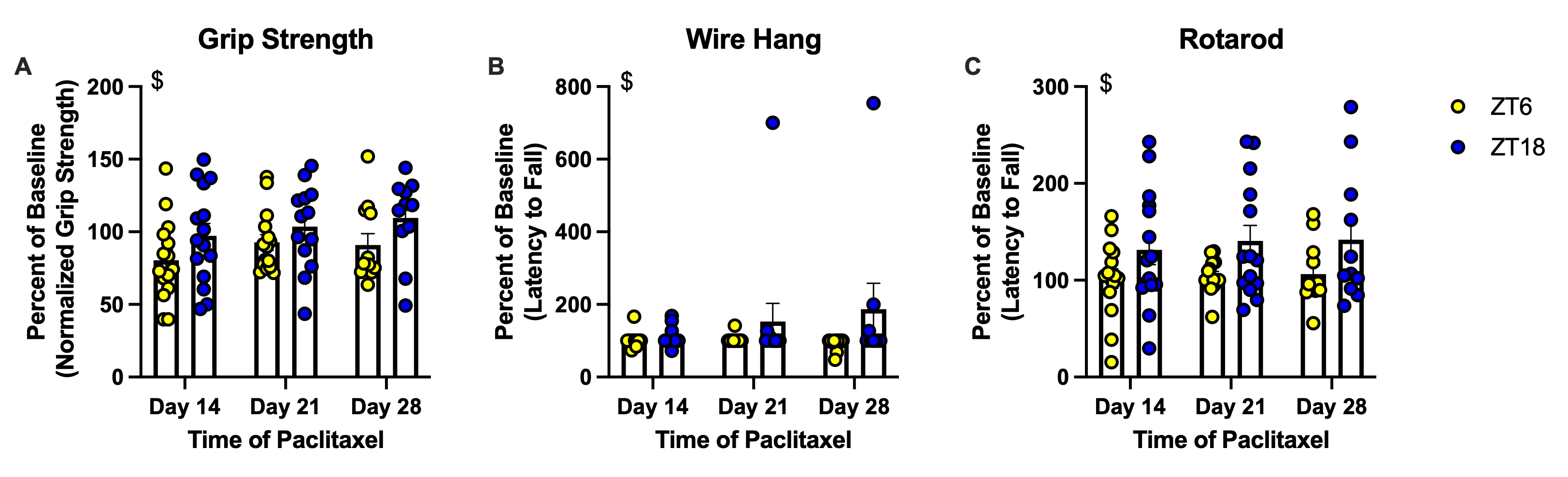
**


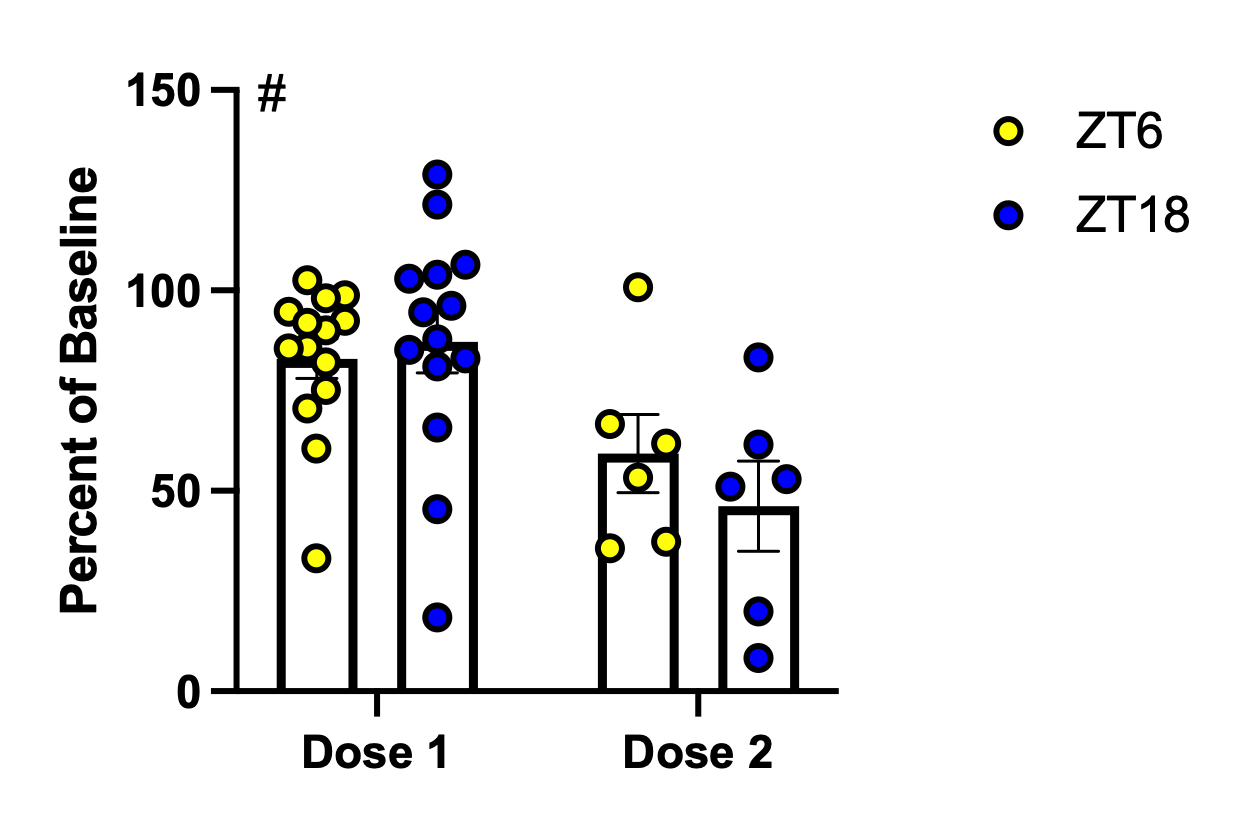


**Figure S2 Paclitaxel induces a fatigue-like phenotype via VWRA.** Total active counts (i.e., wheel revolutions during the active phase) for three 24-hour cycles leading up to the first dose of paclitaxel were used as baseline VWRA. Three 24-hour cycles following the first dose were used to calculate a percentage of baseline. Two 24-hour cycles post dose 2 were compared to two 24-hour cycles prior to the first dose for a percentage of baseline calculation following the second dose. This was due to mice reaching ERC before reaching 3 full cycles post dose 2. Analysis following each dose started 6 hours post-treatment (i.e., at ZT12 for mice treated at ZT6 and at ZT0 the following cycle for mice treated at ZT18) to account for acute drop in activity due to injection. Data are presented as mean ± SEM. Significant effect of # dose; dose 1 n=15/time, dose 2 n=6/time due to technical issues in recording counts.


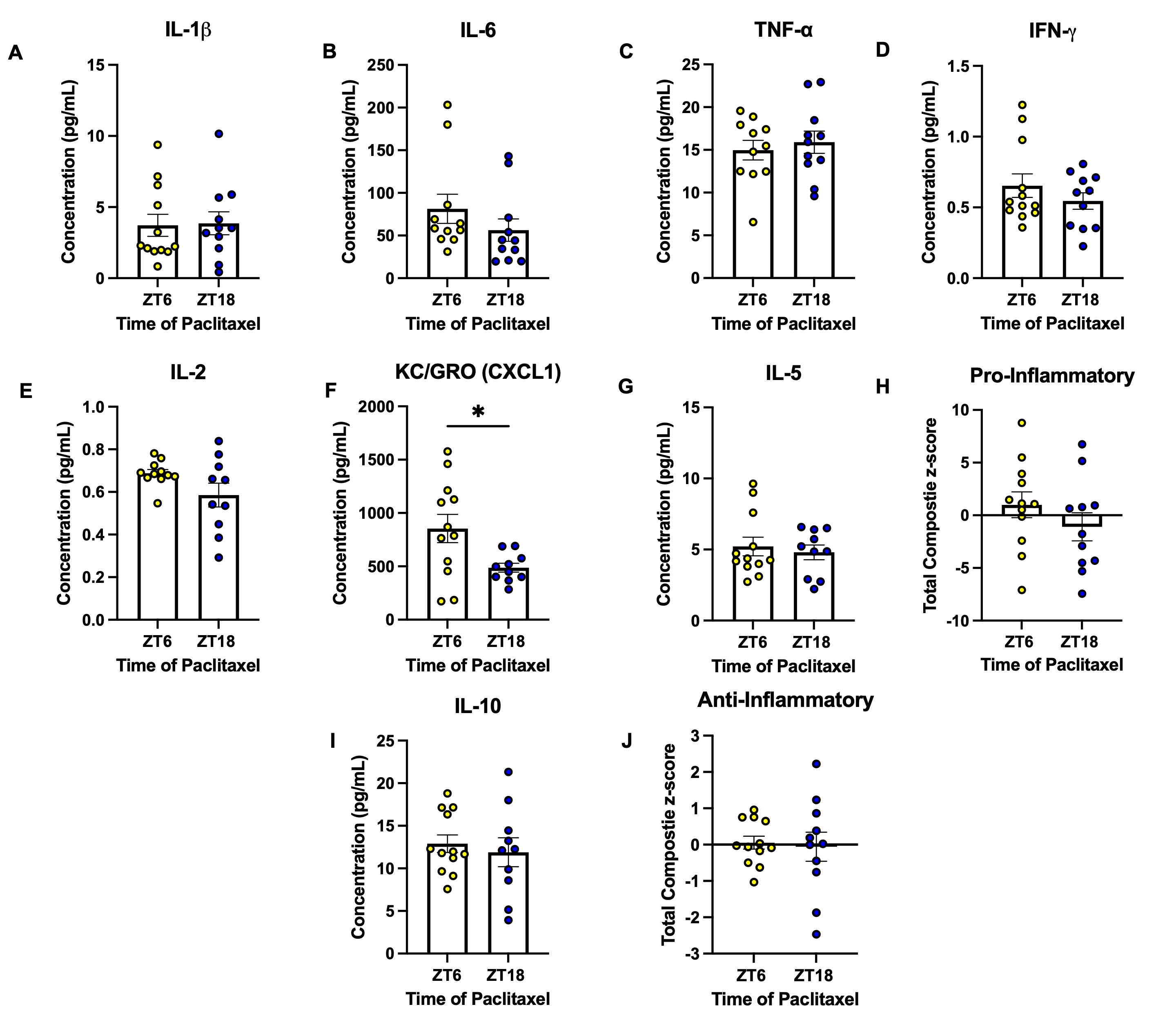


**Figure S3 Circulating concentrations of pro- and anti-inflammatory markers are unaffected by time of treatment with the exception of CXCL1.** Circulating protein levels (serum) of (A) IL-1β, (B) IL-6, (C) TNF-α, (D) IFN-γ, (E) IL-2, (F) CXCL1, (G) IL-5, and (I) IL-10 were measured via ELISA (Mouse pro-inflammatory V-PLEX, MSD). (H) Total inflammatory composite z-score of log-transformed pro-inflammatory cytokines concentrations in circulation. (J) Total inflammatory composite z-score of log-transformed anti-inflammatory cytokines concentrations in circulation. Data are presented as mean ± SEM. *p≤0.05. n=10-12/group.


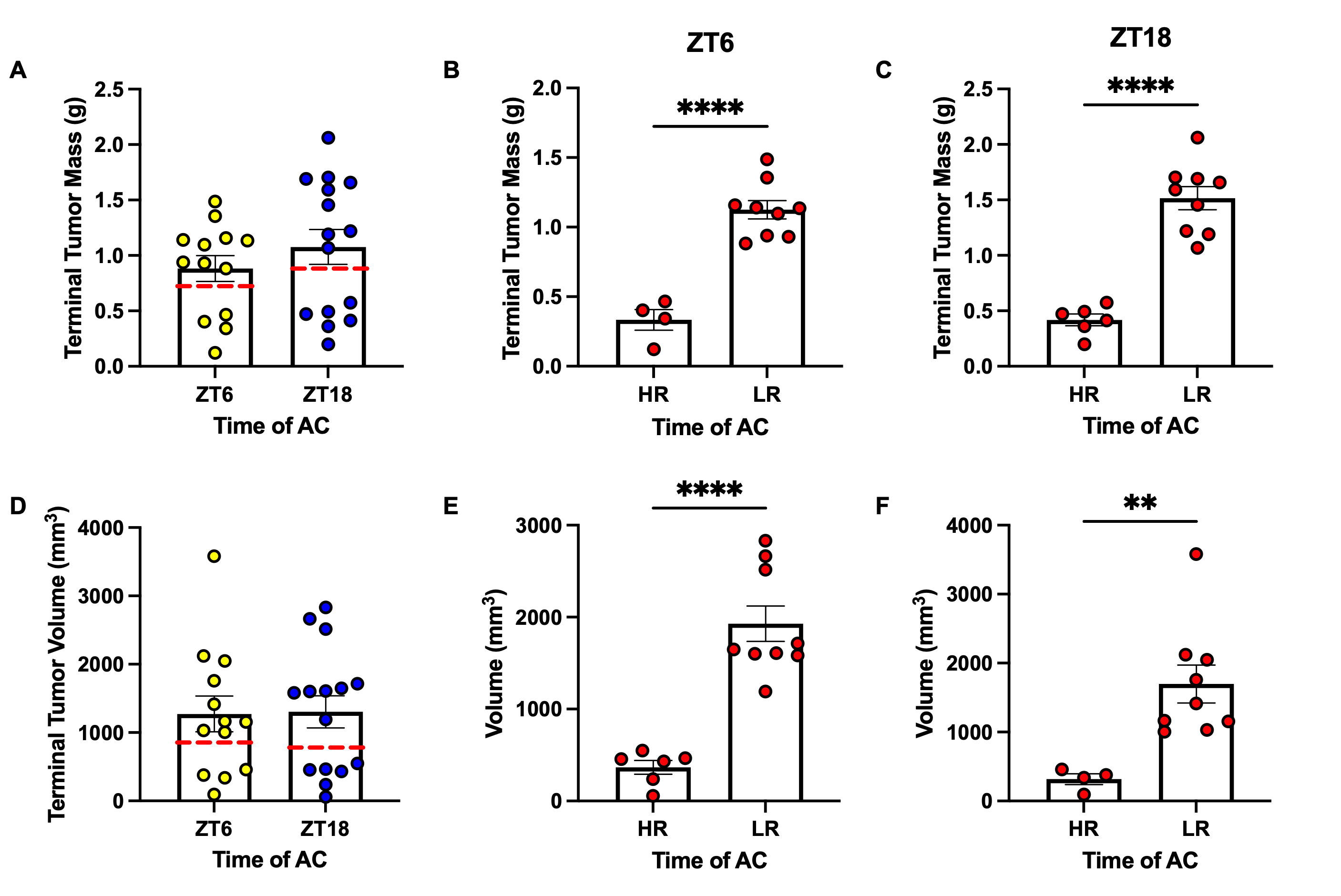


**Figure S4 Mice with a relative low response to AC have significantly elevated tumor masses and volumes.** (A) Terminal tumor mass clusters into two distinct groups based on geometric mean. Mice bearing tumors below the geometric mean (0.7404 g ZT6, 0.8773 g at ZT18) are referred to as having a high response, and those below are referred to as having a low response. Mice treated at both (B) ZT6 and (C) ZT18 with relative low responses to AC had significantly elevated terminal tumor masses. (D) Terminal tumor volumes cluster into the same two distinct groups based on geometric mean (912.2 mm^3^ ZT6, 890.5 mm^3^ ZT18) as they did by mass. Mice treated at both (E) ZT6 and (F) ZT18 with relative low responses to AC had significantly elevated terminal tumor volumes. Data are presented as mean ± SEM. **p≤0.01, **** p≤0.0001. (A,D) n=13-15/group; (B,C,E,F) n=4-9/group.


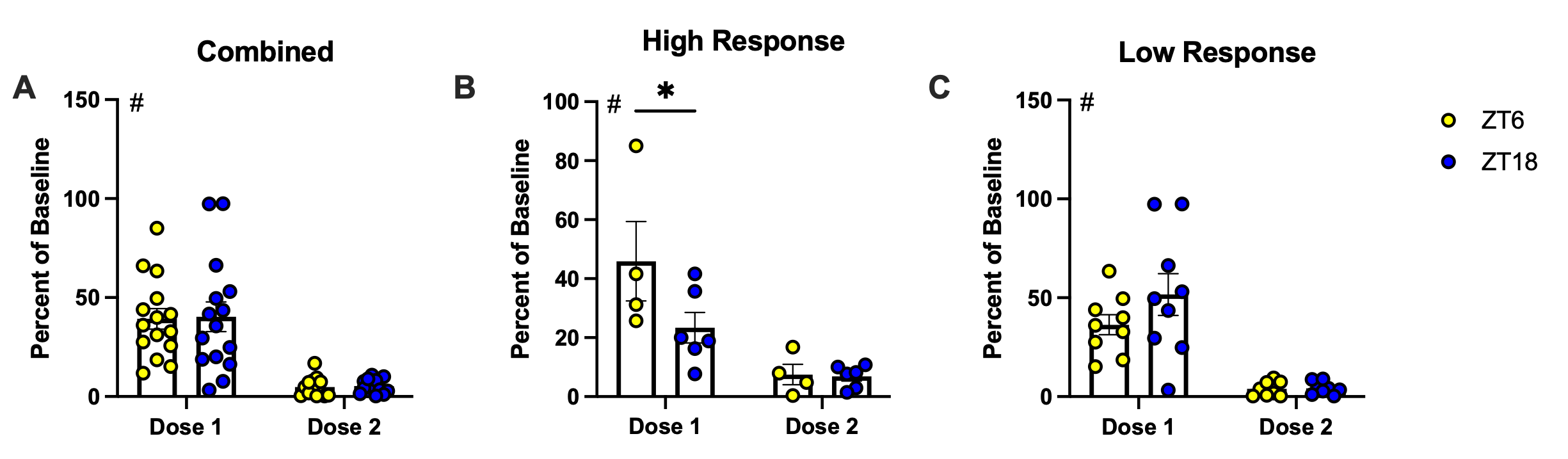


**Figure S5 AC induces a fatigue-like phenotype via VWRA.** Total active counts (i.e., wheel revolutions during the active phase) for three 24-hour cycles leading up to the first dose of AC were used as baseline VWRA. Three 24-hour cycles following each dose were used to calculate a percentage of baseline. (A) Combination of both (B) mice with high response to AC and (C) low response to AC. Analysis following each dose started 6 hours post-treatment (i.e., at ZT12 for mice treated at ZT6 and at ZT0 the following cycle for mice treated at ZT18) to account for acute drop in activity due to injection. Data are presented as mean ± SEM. Significant effect of # dose; (A) n=13-16, (B) n=4-6, (C) n=8-9.


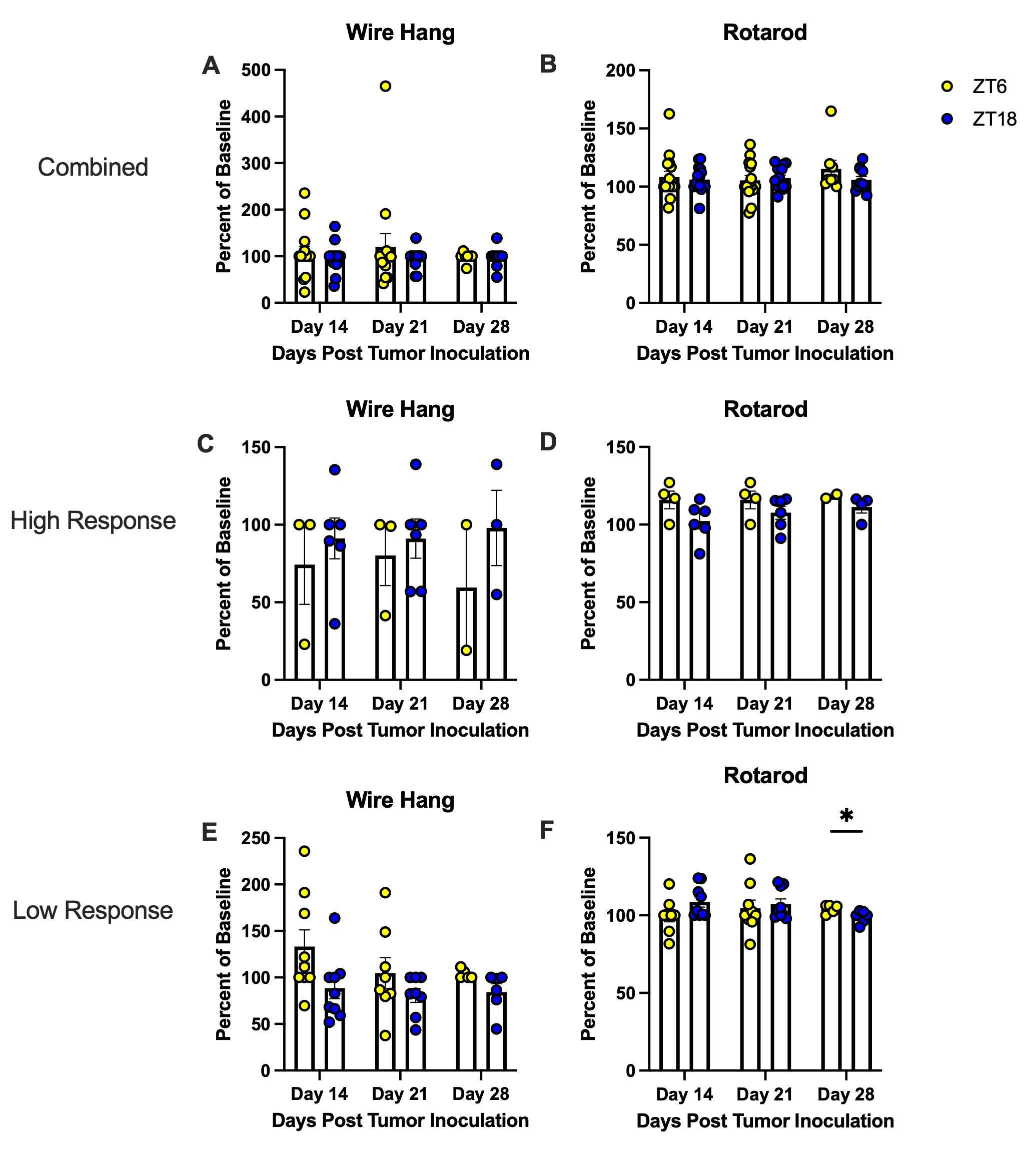


**Figure S6 Latency to fall in wire hang task was unaffected by time of treatment.** Latency to fall from an inverted wire (i.e., wire hang task; a secondary measurement of grip strength) was compared to baseline testing on day 7 (prior to chemotherapy). Percent of baseline latency to fall from the wire in (A) all mice, (C) mice with high response to AC, and (E) mice with low response to AC. Percentage of baseline latency to fall from rotarod (locomotor control) in (B) all mice, (D) mice with high response to AC, and (F) mice with low response to AC. Data are presented as mean ± SEM. *p≤0.05. (A,B) n=7-14, (C,D) n=2-6, (E,F) n=5-9; n varies based on participation in wire hang task and several mice reaching ERC prior to testing on day 28.


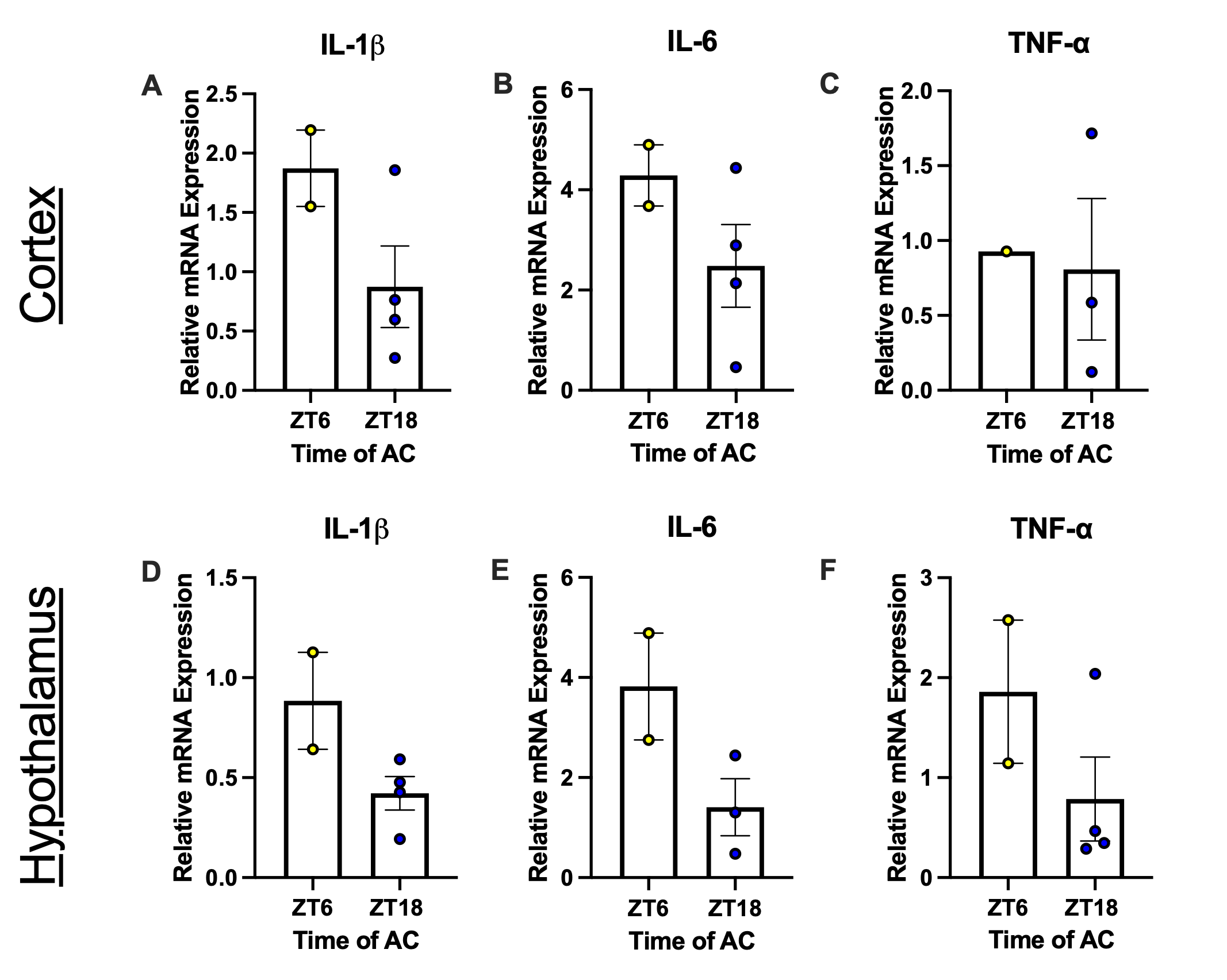


**Figure S7 Pro-inflammatory gene expression in mice with high response to AC.** Relative mRNA expression of cortical (A) IL-1β, (B) IL-6, and (C) TNF-α. Relative mRNA expression of hypothalamic (D) IL-1β, (E) IL-6, and (F) TNF-α. Data are presented as mean ± SEM. n=1-4/group.


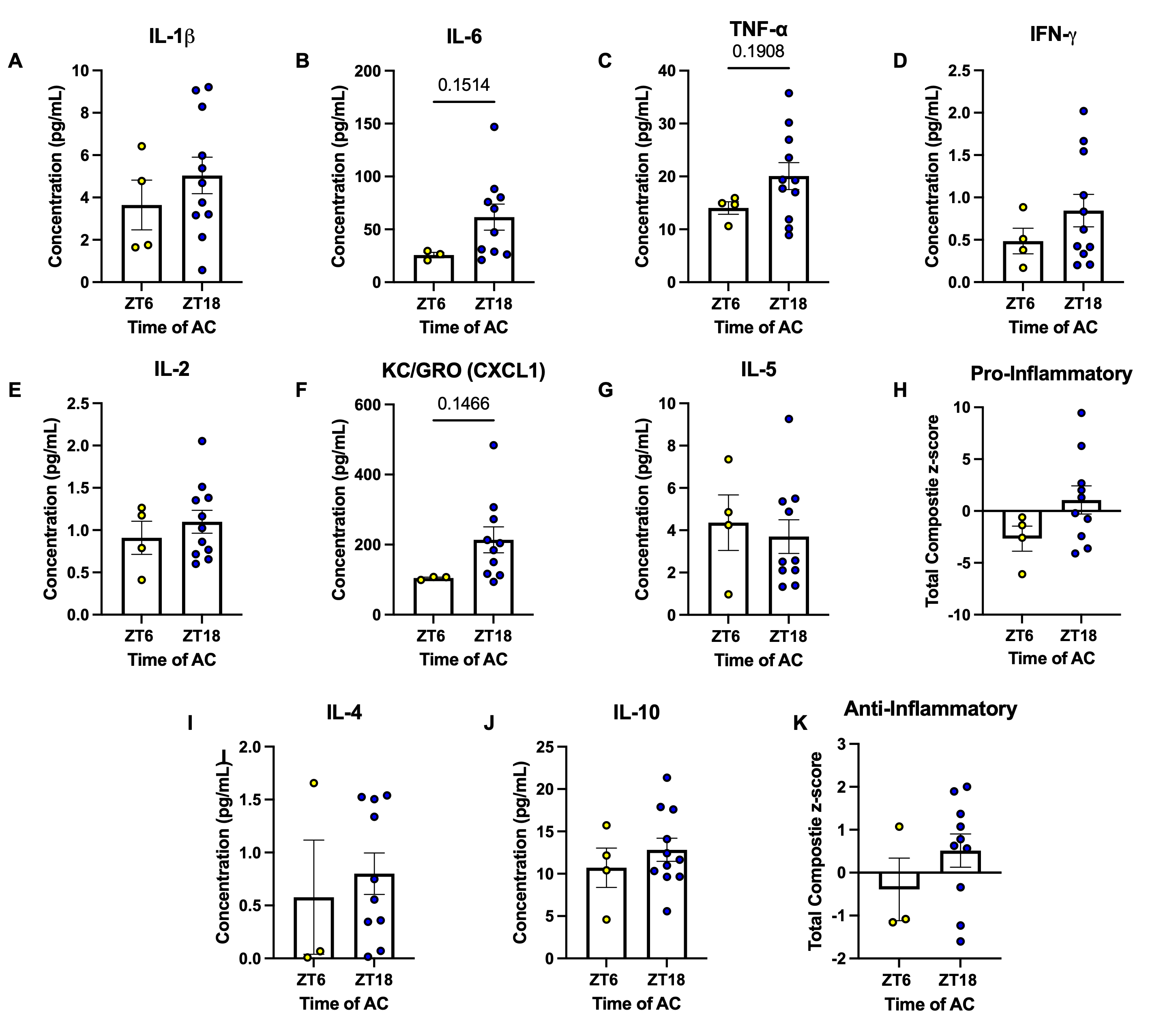


**Figure S8 Circulating concentrations of pro- and anti-inflammatory markers are unaffected by time of treatment.** Circulating protein levels (serum) of (A) IL-1β, (B) IL-6, (C) TNF-α, (D) IFN-γ, (E) IL-2, (F) CXCL1, (G) IL-5, and (I) IL-4, and (J) IL-10 were measured via ELISA (Mouse pro-inflammatory V-PLEX, MSD). (H) Total inflammatory composite z-score of log-transformed pro-inflammatory cytokines concentrations in circulation. (K) Total inflammatory composite z-score of log-transformed anti-inflammatory cytokines concentrations in circulation. Data are presented as mean ± SEM. n=3-10/group.
